# Null Genotypes of *GSTM1* and *GSTT1* and Endometriosis Risk: A Meta-Analysis of 25 Case-Control Studies

**DOI:** 10.1371/journal.pone.0106761

**Published:** 2014-09-10

**Authors:** Haili Zhu, Jiming Bao, Shuguang Liu, Qing Chen, Hong Shen

**Affiliations:** 1 Department of Pathology, Nanfang Hospital, Southern Medical University, Guangzhou, China; 2 Department of Pathology, School of Basic Medical Sciences, Southern Medical University, Guangzhou, China; 3 Department of Urology, Nanfang Hospital, Southern Medical University, Guangzhou, China; 4 Department of Epidemiology, School of Public Health and Tropical Medicine, Southern Medical University, Guangzhou, China; National Cancer Center, Japan

## Abstract

Endometriosis is one of the most frequent benign gynecological disorders. Numerous studies have shown an association between *GSTM1* and/or *GSTT1* polymorphisms and endometriosis susceptibility. However, these associations remain inconclusive. To derive a more precise estimation, we conducted a comprehensive search to identify all existing studies and then performed a meta-analysis. Electronic literature searches of the PubMed, Chinese Biomedical, and China National Knowledge Infrastructure databases were performed up to December 2013. *GSTM1-*, *GSTT1-*, and dual-null genotypes were analyzed independently, and pooled odds ratios (ORs) with 95% confidence intervals (95% CIs) were calculated by comparing the null genotype with other genotypes using the random-effects or fixed-effects model. Twenty-five and 16 independent studies on *GSTM1* and *GSTT1* polymorphisms, respectively, and five *GSTM1-GSTT1* interaction analyses were identified and included in this meta-analysis. Both *GSTM1-* and *GSTT1-*null genotypes increased risk of endometriosis (OR = 1.54, 95% CI: 1.30–1.83, *P*<0.001; OR = 1.41, 95% CI: 1.10–1.82, *P* = 0.007; respectively). Moreover, we found a significant positive association between the dual null genotype *GSTM1-GSTT1* and endometriosis susceptibility (OR = 1.33, 95% CI: 1.03–1.72, *P* = 0.027). This meta-analysis provides evidence that null genotypes of *GSTM1* and/or *GSTT1* contribute to risk of endometriosis. Further investigations are required to confirm these findings.

## Introduction

Endometriosis, a benign gynecological disease, is characterized by the presence of endometrial glands and stroma at extrauterine sites. Approximately 6–10% of women of reproductive age suffer from this condition [1], [2], which negatively impacts quality of life by causing pelvic pain, heavy menstrual flow, dysmenorrhea, and infertility [3], [4], making endometriosis a major public health threat.

The etiology and pathogenesis of endometriosis remain unclear. Endometriosis is commonly considered as a complex trait caused by the interaction between genetic and environmental factors [5]. Both genetic polymorphisms and environmental factors are considered risk factors for endometriosis [6], [7]. Even though no simple Mendelian inheritance was confirmed, geneticfactors increased the risk of endometriosis by 6% for near relatives [8].

Polymorphisms in the glutathione S-transferase (GST) system have long been recognized as a risk factor for endometriosis and have been extensively explored. Human GSTs are a multigene family of phase II metabolizing enzymes that are crucial in detoxification of xenobiotics such as carcinogens, environmental toxins and drugs [9]. Human cytosolic GSTs have been subdivided into eight distinct classes: alpha (GSTA), mu (GSTM), pi (GSTP), theta (GSTT), kappa (GSTK), zeta (GSTZ), omega (GSTO), and sigma (GSTS) [10]. Among the genes in the GST superfamily, *GSTM1* (1p13.3, MIM 138350) [11] and *GSTT1* (22q11.2, MIM 134660) [12] are the most extensively studied owing to their critical role in detoxification and the high-frequency of allelic variants. *GSTM1* and *GSTT1* polymorphisms have been suggested to be associated with endometriosis by many epidemiological studies [13]–[24]. However, findings on the direction of the association remain equivocal.

A previous meta-analysis conducted by Sun-Wei Guo [25] in 2005 indicated a significant association between the *GSTT1*-null genotype and endometriosis, however no such association was found between the *GSTM1-* or *GSTM1-GSTT1*-null genotypes and endometriosis risk. Since then, 12 relevant studies [5], [8], [19]–[24], [26]–[29] have further examined the associations between the two polymorphisms and endometriosis risk. We aimed to confirm the potential associations by conducting an updated meta-analysis, to provide insight into the pathophysiology of endometriosis.

## Methods

### Identification and eligibility of studies

Studies were identified by searching the PubMed, CBM (Chinese Biomedical), and CNKI (China National Knowledge Infrastructure) databases for relevant reports published prior to December 2013, using the key words “*GSTM1*”, “*GSTT1*”, “polymorphisms”, “endometriosis”, and combined phrases. Additional literature was collected from cross-references within both original and review articles. No restrictions on language, population, or sample size were set in this meta-analysis. Studies were required to comply with the following inclusion criteria: (1) original case-control or cohort studies; (2) studies investigated the association of *GSTM1* or *GSTT1* polymorphism with risk of endometriosis; (3) sufficient information to calculate odds ratios (ORs) with 95% confidence intervals (CIs); and (4) Chinese articles were published in Chinese core periodicals. Exclusion criteria were: (1) not case-control or cohort studies evaluating the association of the *GSTM1* or *GSTT1* polymorphism with endometriosis; (2) case reports, letters, reviews, editorials, or correspondence articles; (3) studies based on incomplete raw data; and (4) studies that contained overlapping data.

### Data extraction

The data from eligible studies was independently checked and extracted according to the pre-specified selection criteria, and the discrepancies were resolved by discussion and agreement between all investigators. The following information was collected from each included study: name of the first author, publication year, study location, ethnicity, source of controls, sample-size of cases and controls, and genotype frequency in cases and controls. Different ethnicity descents were categorized as European and Asian. According to source of controls, all included studies were defined as population-based (PB) and hospital-based (HB).

### Quality score assessment

Two investigators independently assessed the quality of included studies using the Newcastle–Ottawa Scale (NOS) [30]. The NOS ranges from zero to nine stars. Studies with a score of seven stars or greater were considered to be of high quality. Discrepancies were resolved as described above.

### Statistical analysis

Meta-analyses were performed for polymorphisms investigated in at least three studies [31], [32]. The strength of the associations between *GSTM1* and *GSTT1* polymorphisms and endometriosis risk were measured by ORs and respective 95% CIs, which were calculated by comparing the null genotype with other genotypes. The significance of the pooled ORs was determined by the Z-test (*P*<0.05 was considered significant). Subgroup analyses were performed by ethnicity and source of controls.

Heterogeneity among studies in terms of degree of association was evaluated using χ^2^ tests. The I^2^ statistic was used to evaluate variations due to heterogeneity rather than chance. *P*<0.10 or I^2^>50% indicated the presence of between-study heterogeneity, and the random-effects model (DerSimonian-Laird method) [33] was used to calculate the pooled ORs; otherwise, the fixed- effects model (Mantel-Haenszel method) [34] was selected.

Sensitivity analyses were performed to evaluate the stability of the results of the meta-analysis. The influence of individual studies was evaluated by estimating the pooled ORs in the absence of each study [35]. Potential publication bias was investigated by visual inspection of Begg’s funnel plots. We also used the Begg’s [36] and Egger’s tests [37] to evaluate any possible publication bias (*P*<0.05 was treated as significant publication bias). All statistical analyses were performed using Stata statistical software, version 12.0 (Stata Corp., College Station, TX, USA).

## Results

### Study selection and characteristics

**Figure 1:**
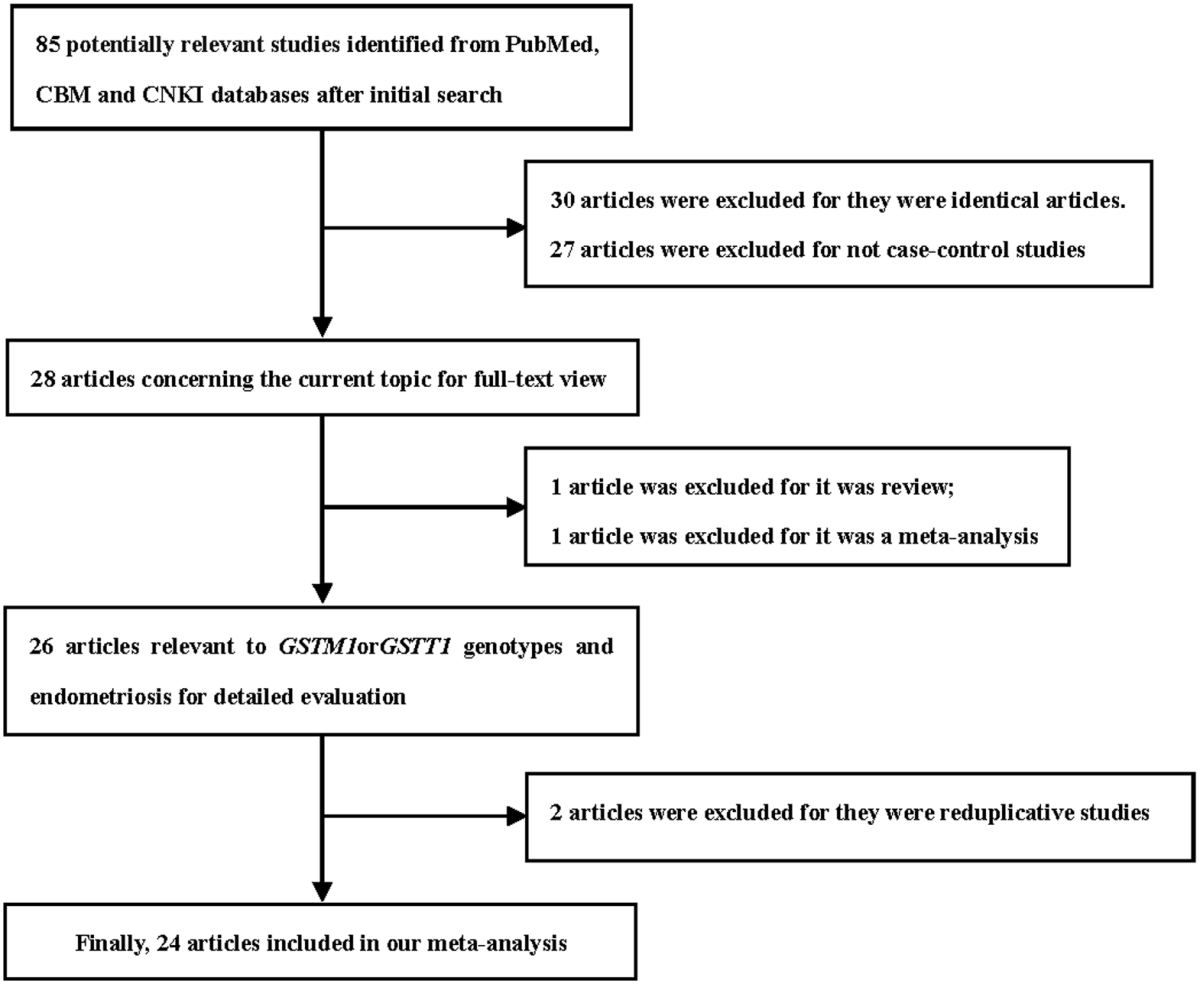
Flow diagram of the study selection process. CBM: Chinese Biomedical; CNKI: China National Knowledge Infrastructure.

This meta-analysis is guided by the PRISMA statement (**Checklist S1**). We initially identified 85 results relevant to the search terms in the selected databases. After reading the titles and abstracts, only 28 potentially eligible articles were identified for further detailed evaluation. Of these, two articles [38], [25] were excluded because one article was a review and the other was a meta-analysis. After further screening, two further articles [39], [40] were excluded for overlapping populations. Two ethnic populations (Hans and Uyghurs) were studied in the article by Ding et al [18], so we considered it as two studies in this analysis. In total, we included 24 articles [5], [8], [13]–[24], [26]–[29], [41]–[46], including 25 independent case-control studies (**Figure 1**). Eighteen, four, and two of the articles were written in English, Chinese, and Russian, respectively. Twenty-five and 16 studies focused on *GSTM1* and *GSTT1* polymorphisms, respectively, and five were on *GSTM1-GSTT1* interaction analysis. The characteristics of the included studies are described in **Table 1**.

**Table 1:** Characteristics of studies included in the meta-analysis.

First author[reference]	Year	Country	Ethnicity	Source ofControls	Case	Control	Null *GSTM1*Case Control	Null *GSTT1*Case Control	Dual nullCase Control	Qualityscore
Baranov et al. [13]	1996	Russia	European	PB	42	67	34 26	0 0	0 0	7
Baranov et al. [41]	1999	Russia	European	PB	150	99	88 42	0 0	0 0	7
Baranova et al. [14]	1999	France	European	HB	65	72	50 33	13 7	0 0	7
Baxter et al. [42]	2001	England	European	PB	84	219	40 107	0 0	0 0	7
Hadfield et al. [43]	2001	England	European	HB	132	52	59 27	29 14	0 0	8
Ivashchenko et al. [44]	2003	Russia	European	HB	74	39	42 17	27 6	0 0	8
Arvanitis et al. [15]	2003	Greece	European	HB	275	346	161 181	24 31	11 16	8
Peng et al. [16]	2003	China	Asian	HB	76	80	50 37	0 0	0 0	6
Lin et al. [17]	2003	China	Asian	HB	68	28	49 12	53 9	0 0	7
Morizane et al. [45]	2004	Japan	Asian	PB	108	173	57 89	52 71	30 43	9
Ding (a) et al. [18]	2004	China	Asian	HB	41	107	21 57	15 32	0 0	8
Ding (b) et al. [18]	2004	China	Asian	HB	80	105	46 55	59 47	0 0	8
Hur et al. [46]	2005	Korea	Asian	HB	194	259	112 145	104 125	132 154	7
Babu et al. [19]	2005	India	Asian	PB	310	215	121 64	42 34	14 11	8
Aban et al. [20]	2007	Turkey	European	HB	150	150	88 65	59 44	0 0	8
Kim et al. [26]	2007	Korea	Asian	HB	316	256	183 146	178 124	0 0	7
Yang et al. [21]	2009	China	Asian	HB	216	216	134 100	0 0	0 0	7
Cao et al. [27]	2009	China	Asian	HB	51	102	33 61	22 39	0 0	7
Roya et al. [22]	2009	India	Asian	HB	97	102	26 15	0 0	0 0	6
Huang et al. [28]	2010	China	Asian	HB	28	29	12 10	0 0	0 0	7
Hosseinzadeh et al. [23]	2011	Iran	Asian	HB	120	200	87 80	0 0	0 0	5
Trabert et al. [5]	2011	USA	European	PB	254	567	137 268	0 0	0 0	9
Wu et al. [24]	2012	China	Asian	HB	121	171	57 52	40 33	23 15	7
Vichi et al. [8]	2012	Italy	European	HB	181	162	104 85	20 32	0 0	8
Matsuzaka et al. [29]	2012	Japan	Asian	HB	97	143	43 67	38 56	0 0	7

Abbreviations: PB, population-based; HB, hospital-based.

### Quality assessment results

The scores of included studies were 5 to 9 (**Table 1**).

### Meta-analysis results

**Figure 2:**
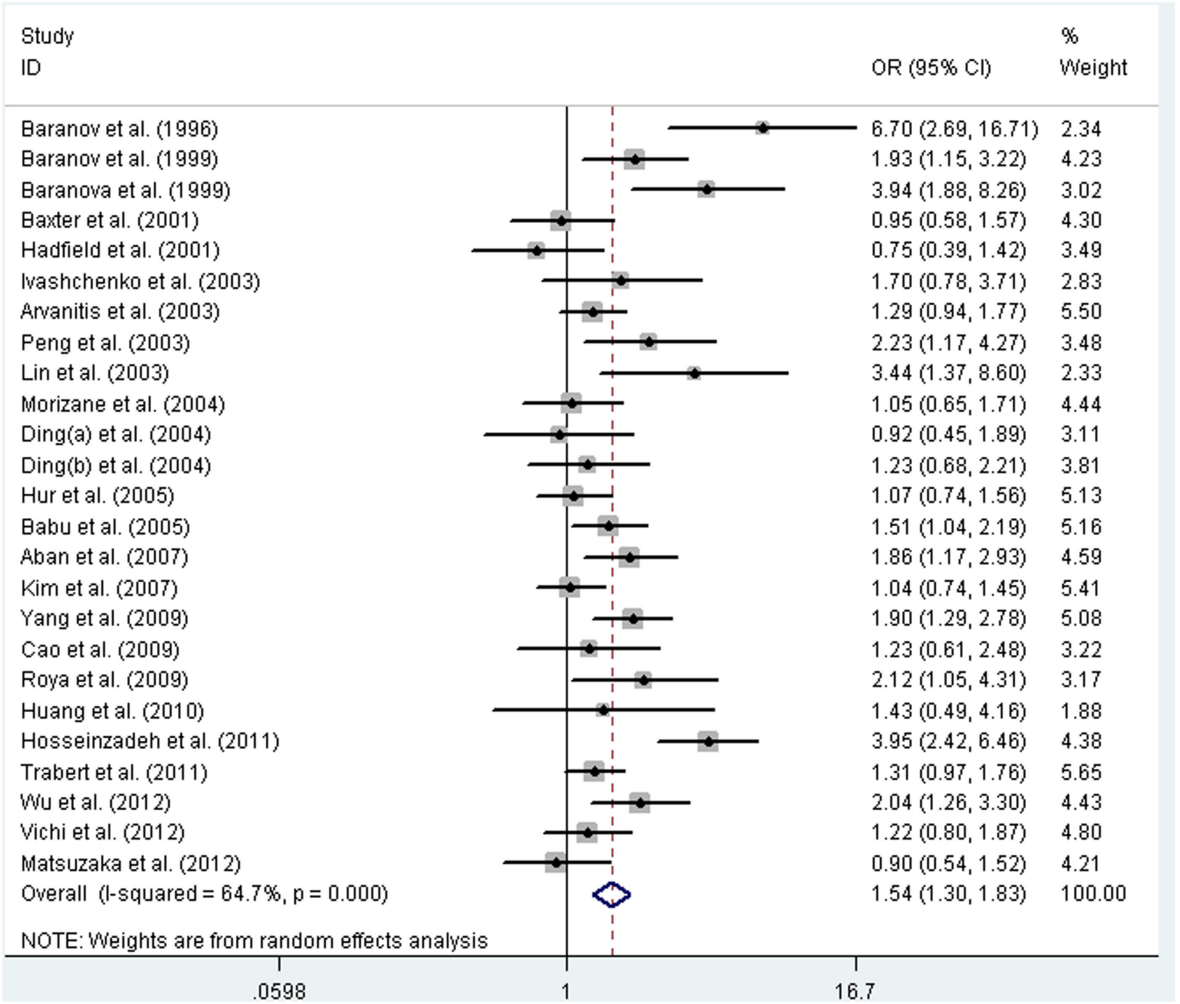
Forest plot of pooled OR with 95% CI for association between the null genotype of *GSTM1* and endometriosis risk.

The association between *GSTM1* polymorphism and endometriosis was investigated in 25 studies, which included a total of 3330 cases and 3959 controls. The heterogeneity was significant, so the random-effects model was selected. The result showed that the null genotype of *GSTM1* was associated with an increased endometriosis risk (OR = 1.54, 95% CI: 1.30–1.83, *P*<0.001). A forest plot is shown in **Figure 2**. Furthermore, we included 22 studies of high quality to validate the association, and the result again showed a strong association between this polymorphism and endometriosis risk (OR = 1.45, 95% CI: 1.23–1.71, *P*<0.001). Subgroup analysis by ethnicity was performed, and an increased risk of endometriosis was observed in Europeans and Asians (OR = 1.58, 95% CI: 1.19–2.09, *P* = 0.002; OR = 1.52, 95% CI: 1.21–1.91, *P*<0.001; respectively). In stratified analysis by source of controls, the results showed that source of controls did not affect the pooled results and a significantly increased risk of endometriosis was detected both in PB and HB studies (OR = 1.52, 95% CI: 1.08–2.16, *P* = 0.005; OR = 1.55, 95% CI: 1.26–1.91, *P*<0.001; respectively).

**Figure 3:**
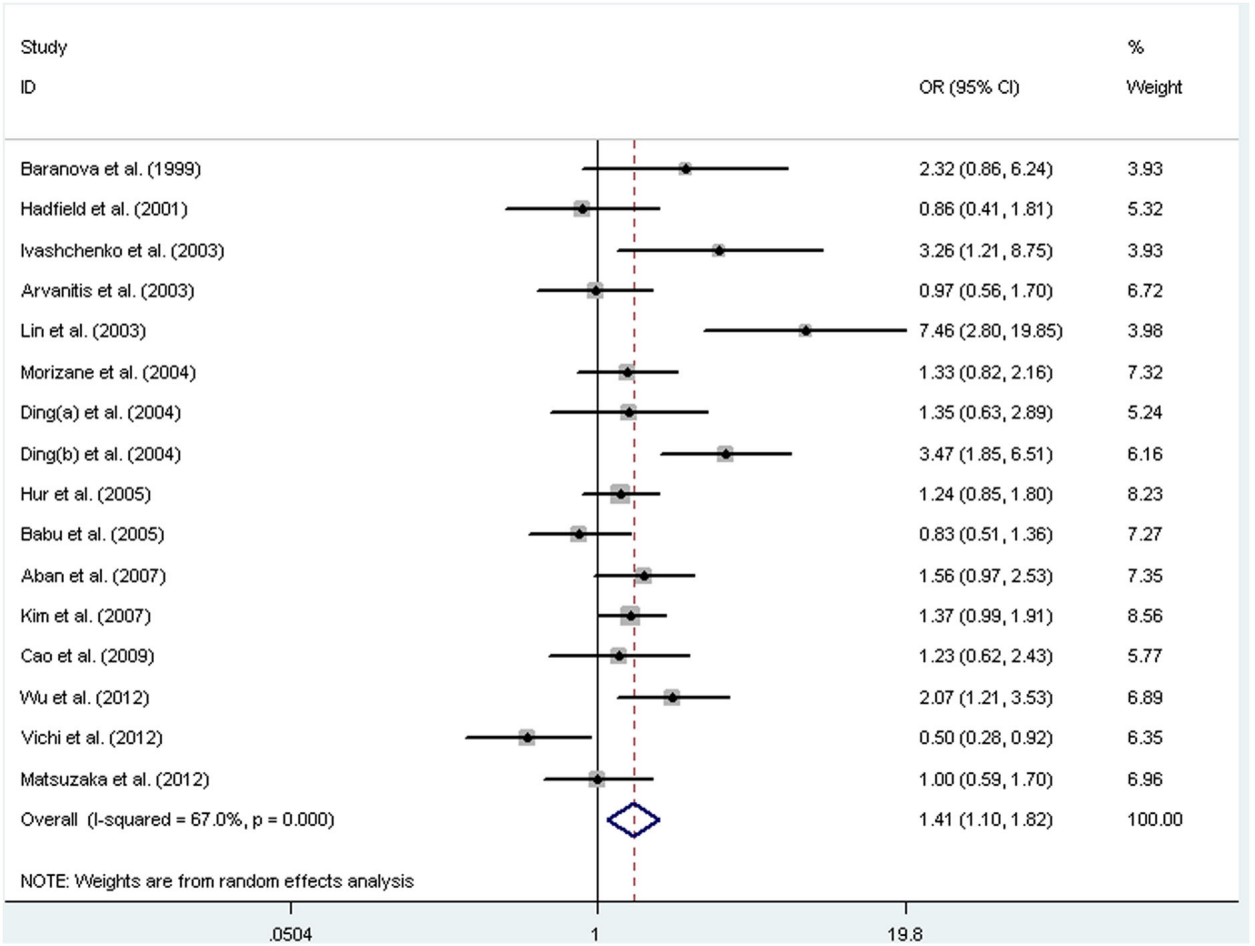
Forest plot of pooled OR with 95% CI for association between the null genotype of *GSTT1* and endometriosis risk.

Sixteen independent studies, with a total of 2263 cases and 2380 controls, were included in the meta-analysis of *GSTT1* polymorphism. We found significant heterogeneity between studies, so the random-effects model was used to pool the results. The results indicated a positive association between the null genotype of *GSTT1* with endometriosis risk (OR = 1.41, 95% CI: 1.10–1.82, *P* = 0.007). A forest plot is shown in **Figure 3**. Stratified analysis by ethnicity showed a significant association in Asians (OR = 1.53, 95% CI: 1.14–2.06, *P* = 0.005), but not in Europeans (OR = 1.21, 95% CI: 0.74–1.99, *P* = 0.45).

**Figure 4:**
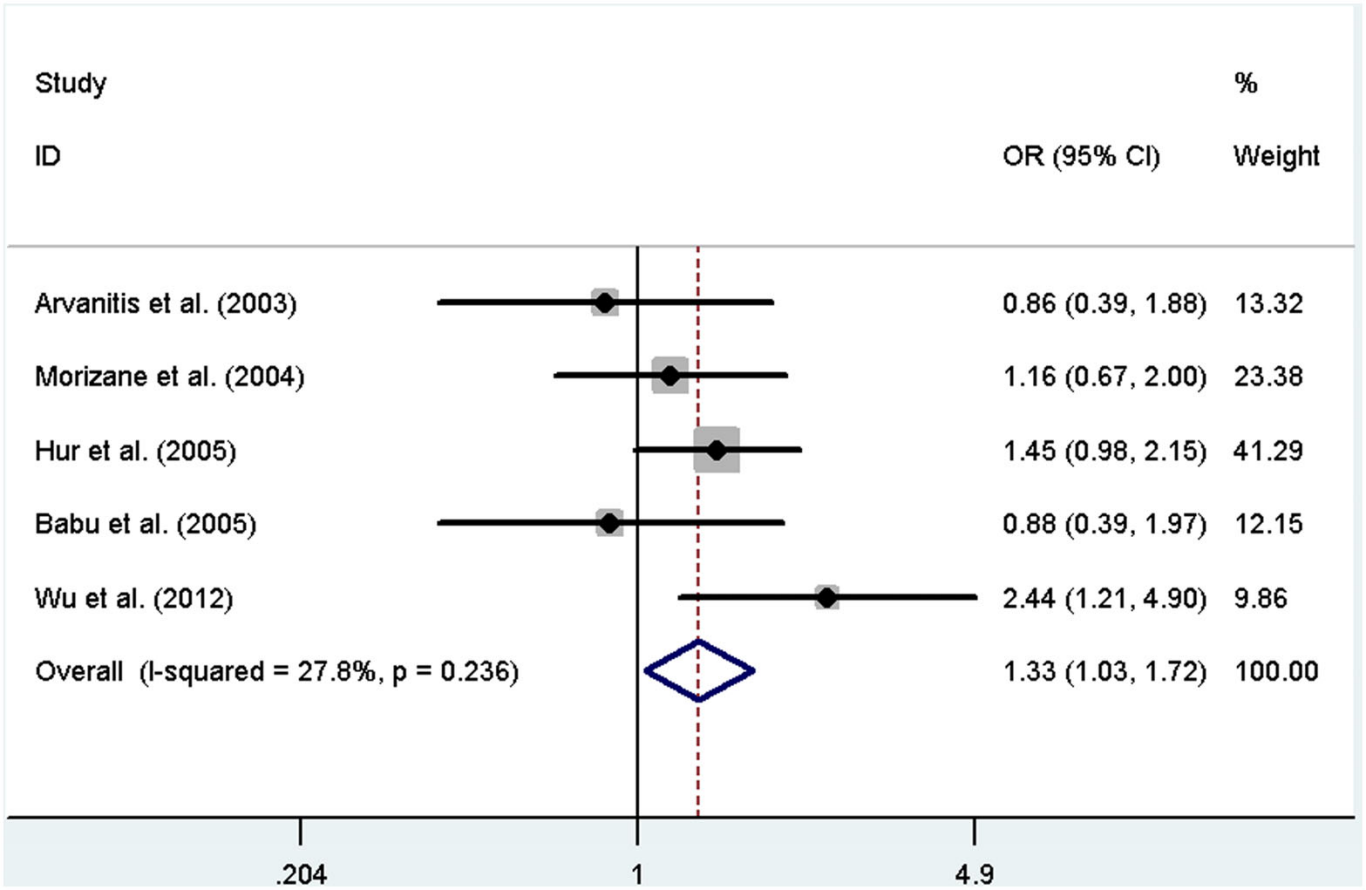
Forest plot of pooled OR with 95% CI for association between the dual null genotype of *GSTM1*-*GSTT1* and endometriosis risk.

For *GSTM1-GSTT1* interaction analysis, five independent studies, including 1008 cases and 1164 controls, were subjected to meta-analysis. The heterogeneity was not significant, so we selected the fixed-effects model. The results showed that the dual null genotype of *GSTM1-GSTT1* was associated with an increased endometriosis risk (OR = 1.33, 95% CI: 1.03–1.72, *P* = 0.027). A forest plot is shown in **Figure 4**. The results of this meta-analysis are summarized in **Table 2**.

**Table 2:** Meta-analysis of associations between *GSTM1*, *GSTT1,* and *GSTM1-GSTT1* polymorphisms and endometriosis.

Genes	Comparisons	Sample size Case Control		No.ofstudies	Test of association OR (95% CI) *p* Model				Test of heterogeneity χ^2^ *p* I^2^		
*GSTM1*											
Overall	*GSTM1*(−) vs. *GSTM1*(+)	3330	3959	25	1.54	1.30–1.83	<0.001	R	0.115	<0.001	64.7
BS	*GSTM1*(−) vs. *GSTM1*(+)	3037	3577	22	1.45	1.23–1.71	<0.001	R	0.080	<0.001	57.3
Ethnicity											
European	*GSTM1*(−) vs. *GSTM1*(+)	1407	1773	10	1.58	1.19–2.09	0.002	R	0.130	0.001	68.5
Asian	*GSTM1*(−) vs. *GSTM1*(+)	1923	2186	15	1.52	1.21–1.91	<0.001	R	0.121	<0.001	64.5
Source of Controls											
PB	*GSTM1*(−) vs. *GSTM1*(+)	948	1340	6	1.52	1.08–2.16	0.005	R	0.124	0.018	70.1
HB	*GSTM1*(−) vs. *GSTM1*(+)	2382	2619	19	1.55	1.26–1.91	<0.001	R	0.125	<0.001	64.8
*GSTT1*											
Overall	*GSTT1*(−)vs. *GSTT1*(+)	2263	2380	16	1.41	1.10–1.82	0.007	R	0.163	<0.001	67.0
Ethnicity											
European	*GSTT1*(−) vs. *GSTT1*(+)	877	821	6	1.21	0.74–1.99	0.450	R	0.253	0.007	68.4
Asian	*GSTT1*(−) vs. *GSTT1*(+)	1386	1559	10	1.53	1.14–2.06	0.005	R	0.143	0.001	67.5
*GSTM1-GSTT1*	*GSTM1-GSTT1*(−) vs.other genotypes	1008	1164	5	1.33	1.03–1.72	0.027	F	0.037	0.236	27.8

Abbreviations: OR, odds ratio; CI, confidence interval; BS, based on score (studies with quality scores <7 were excluded); PB, population-based; HB, hospital-based; F, fixed-effects model; R, random-effects model.

### Sensitivity analysis

**Figure 5:**
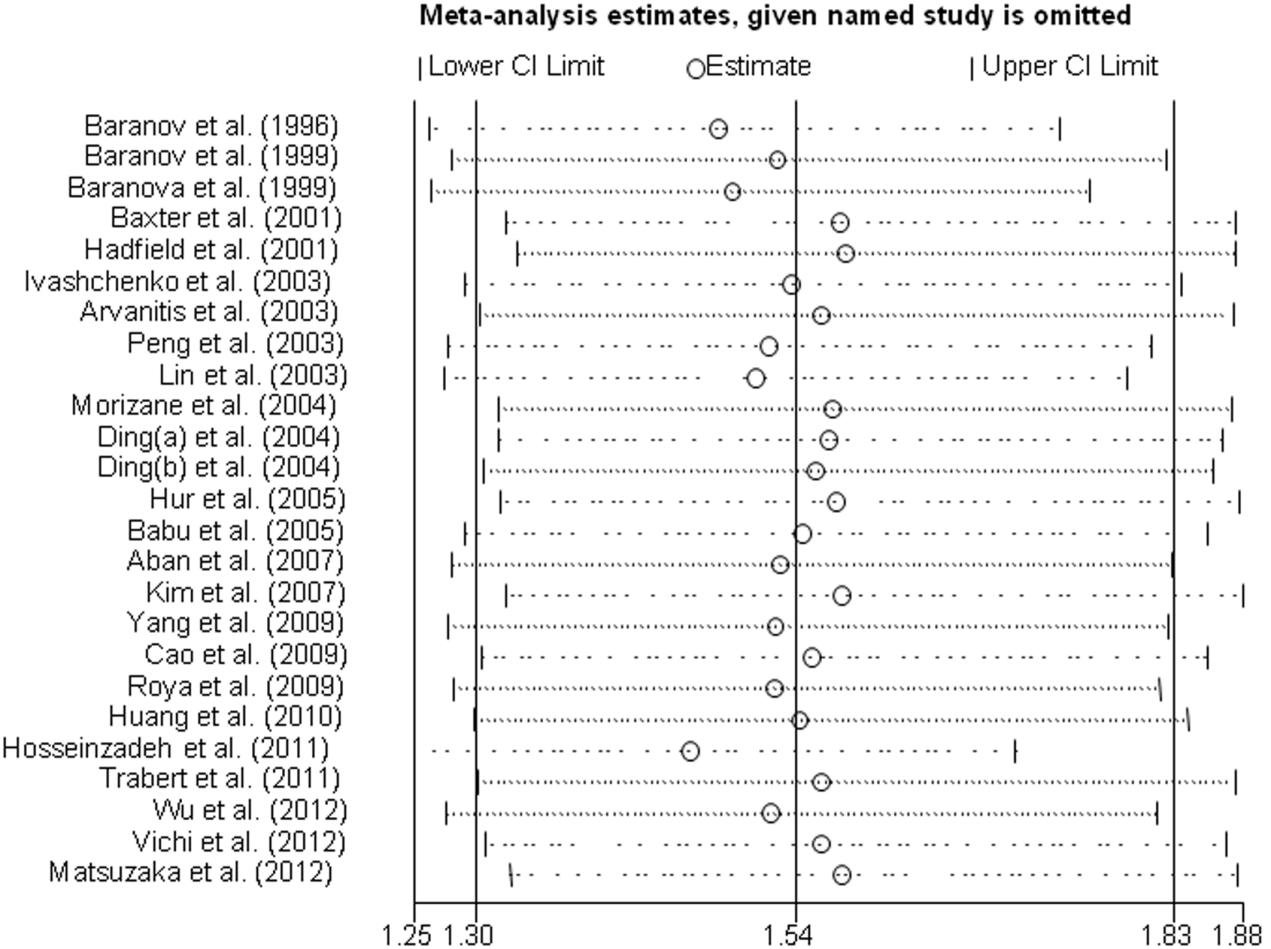
Sensitivity analysis for the meta-analysis regarding the association between *GSTM1* polymorphism and endometriosis risk.

Sensitivity analyses were performed after the sequential removal of each eligible study to assess the influence of each individual study on the pooled ORs. In the analysis of the *GSTM1* polymorphism, the pooled ORs were not qualitatively changed when any single study was omitted, suggesting that no single study exhibited excessive influence, and that the results are reliable (**Figure 5**). Other results were also relatively stable.

### Publication Bias

**Figure 6:**
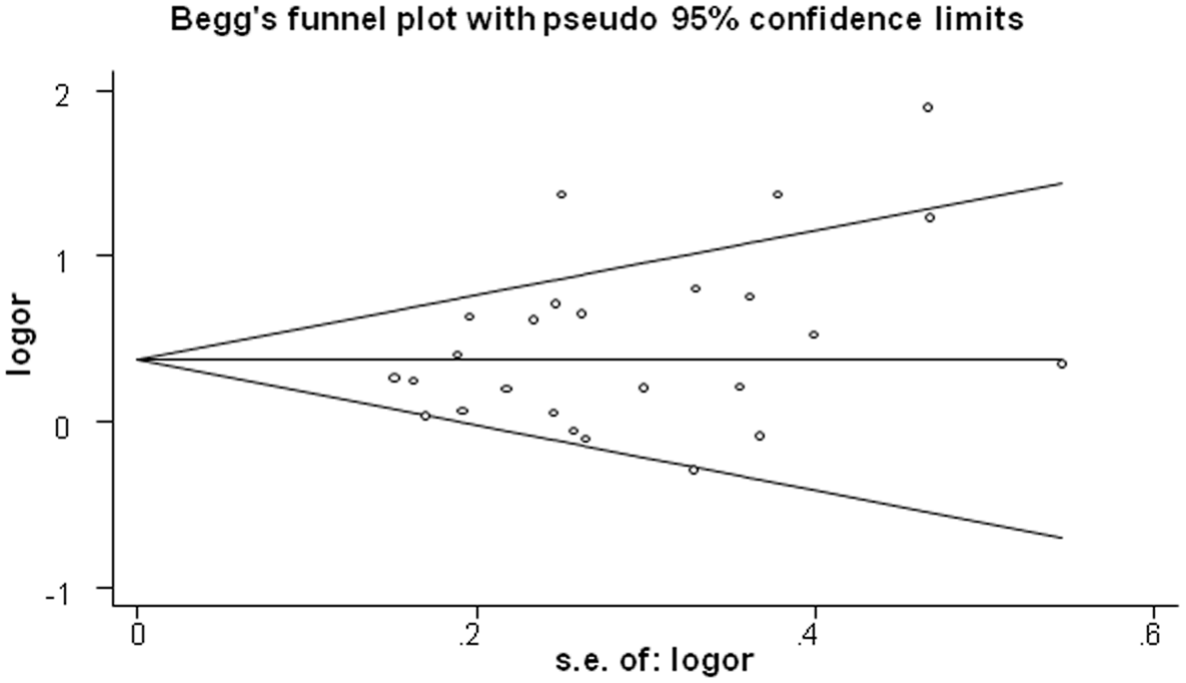
Begg’s funnel plot for publication bias in selection of studies regarding the *GSTM1* polymorphism.

**Figure 7:**
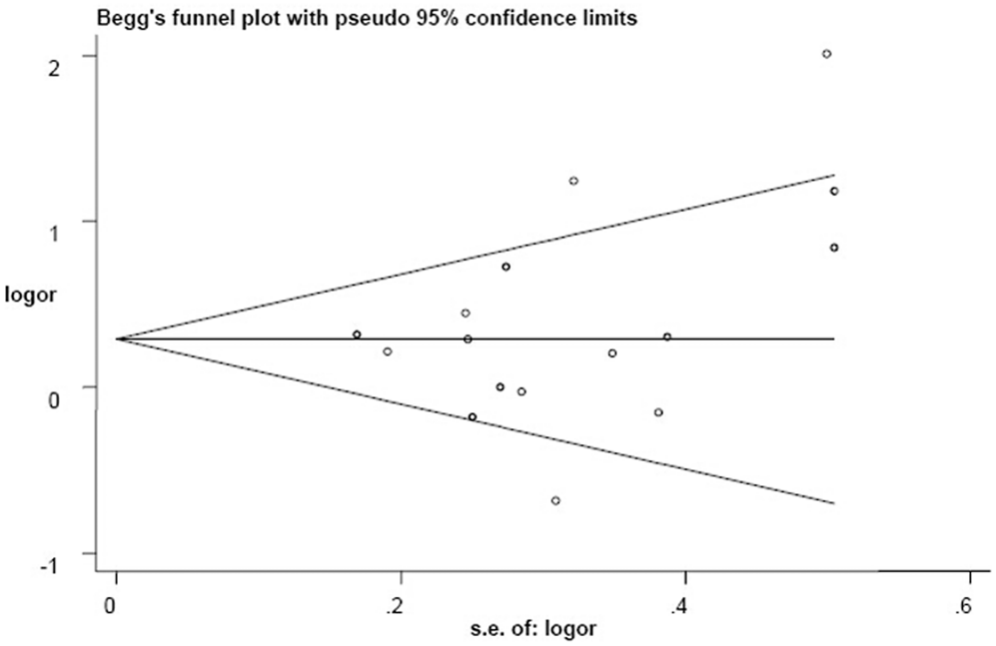
Begg’s funnel plot for publication bias in selection of studies regarding the *GSTT1* polymorphism.

We conducted Begg’s and Egger’s tests to evaluate potential publication bias. There was no statistical evidence of publication bias regarding analysis of the *GSTM1* polymorphism (*GSTM1* (−) vs. *GSTM1* (+): Begg, *P* = 0.129 and Egger, *P* = 0.079), and Begg’s funnel plots suggested no substantial asymmetry (**Figure 6**). There was no publication bias for other results (*GSTT1* (−) vs. *GSTT1* (+): Begg, *P* = 0.344 and Egger, *P* = 0.207, and the shape of the funnel plot also did not reveal any evidence of obvious asymmetry (**Figure 7**); *GSTM1-GSTT1* (−) vs. other genotypes: Begg, *P* = 0.462 and Egger, *P* = 0.613).

## Discussion

As many GST genes are polymorphic, whether particular allelic variants in GST genes are correlated with altered risk of some kinds of diseases has provoked great interest [47]. The null genotypes of *GSTM1* and *GSTT1*, two of the most widely-studied polymorphisms, are characterized by homozygous deletions of the respective genes [39]. The first study considering null genotype of *GSTM1* as risk factors was conducted by Baranov et al. [13] in 1996. A preponderance of *GSTT1*-null subjects among endometriosis patients was detected in 1999, although it was not statistically significant [14]. Since then, many studies have investigated the associations between null genotypes of *GSTM1* and *GSTT1* and endometriosis susceptibility. However, the results are inconsistent and conflict, which compelled us to pay attention to the two polymorphisms at a meta-analytical level.

The current study, including 25 case-control studies with 3330 cases and 3959 controls, is the most comprehensive meta-analysis of the association of *GSTM1-* and *GSTT1*-null genotypes with endometriosis risk, which allows us to expand the discussion of possible implications and interpretations of the findings. Our meta-analysis suggested that there were significant associations between the null genotypes of *GSTM1* and *GSTT1* and endometriosis. Moreover, the combined *GSTM1-GSTT1*-null genotype also showed a positive association with endometriosis susceptibility.

A novel finding of the present study was the significant positive association between the null genotype of *GSTM1* and endometriosis risk. The large number of studies and subjects included in this meta-analysis were substantial enough to resolve the issue of conflicting results obtained in individual studies, which was primarily caused by their small sample size. Furthermore, the results in subgroup analysis by ethnicity also indicated an association in both Europeans and Asians suggesting this association is reliable.

This meta-analysis showed a moderate positive association between the null genotype of *GSTT1* and endometriosis risk, which is consistent with the result of a previous meta-analysis [25]. In the analysis stratified by ethnicity, a significant association was found in Asians, but no such association was detected among Europeans. There are several possible reasons for such a difference. First, the frequencies of the risk-associated homozygous null genotype vary between different races. The frequency of the *GSTT1*-null genotype is nearly 50% in the Chinese population [48], [49] 14.5–20.1% in Indians [50]–[52], and 11.0–37.9% in Europeans [53], [54]. Thus, the *GSTT1* polymorphism may exert varying effects in different populations. Second, the different results could also be explained by study design or sample size. Other confounding factors, such as age and lifestyle may also be considered.

The *GSTM1-GSTT1* interaction analysis indicated that women with double-null genotype had significantly increased endometriosis risk compared with those with other genotypes. If genetic susceptibility to endometriosis is, at least in part, mediated through polymorphisms of genes that encode enzymes responsible for detoxification, it is possible that the combination of *GSTM1-* and *GSTT1*-null genotypes may be more discriminating as a risk factor for endometriosis than a single null genotype.

The observation that the null genotypes of *GSTM1*, *GSTT1*, and *GSTM1-GSTT1* increased the risk of developing endometriosis is biologically plausible. Environmental contaminants, such as polychlorinated dibenzo-p-dioxins, polychlorinated biphenyls, and polycyclic aromatic hydrocarbon, have been suggested to promote the occurrence and development of endometriosis by interfering with the estrogen signaling pathway and their immunosuppressive effects [55]. The GSTs plays a critical role in the detoxification of a broad range of environmental contaminants. As critical phase II metabolic enzymes, GSTs catalyze reactions between glutathione and all kinds of potentially lipophilic compounds, causing neutralization of the carcinogens, products of oxidative stress and toxic compounds [56]. Previous studies suggested that homozygous null deletions in *GSTM1* and *GSTT1* cause a complete loss of the activity of their encoded enzymes [57], [58]. The *GSTM1* and *GSTT1* deletions are detected in 42–60% and 13–26% of Caucasians, respectively [59]. Lack of GST enzyme activity resulting from the null genotypes may affect the detoxification of environmental toxins, and thus contribute to the pathogenesis of endometriosis.

Heterogeneity is an important issue in meta-analysis. Although we minimized the likelihood by performing a careful search for published studies, using the explicit criteria for study inclusion, statistically significant heterogeneity still existed in most comparisons. There are several explanations for the significant between-study heterogeneity, such as different study populations, genetic factors, and environmental factors. In particular, environmental contaminant exposure, a risk factor for endometriosis, is an important factor contributing to heterogeneity. The status of environmental contamination varies between countries, so the endometriosis incidence varies between populations. Publication bias is another important issue which should also be accounted for in meta-analyses. After evaluating the publication bias using Begg’s funnel plots we did not detect a publication bias, indicating the strength of the results.

Sun-Wei Guo [25] also evaluated the association between *GSTM1* and *GSTT1* polymorphisms and endometriosis risk by performing a meta-analysis including 14 studies with 1539 cases and 1805 controls. That study suggested that the endometriosis risk associated with the null genotype of *GSTT1* was 29% higher than other genotypes, but it failed to find positive associations between the null genotype of *GSTM1* or *GSTM1-GSTT1* and the risk of endometriosis. There were some differences between that study and ours. First, our meta-analysis provided more comprehensive information on the relationships between the two polymorphisms and endometriosis by extracting data from more studies with more total cases and controls. Second, some issues that may affect the results of meta-analysis, such as publication bias, sensitivity analysis, and quality assessment of the included studies, were addressed in our study. Third, the current study also showed distinct findings, with the added statistical power.

This meta-analysis had several limitations that should be taken into account when considering its contributions. First, heterogeneity among studies existed in some comparisons of polymorphisms. Second, the results of our meta-analysis were applicable to only two ethnic groups, Europeans and Asians, as there were no relevant studies including data from African ethnic groups. Hence, to conduct a more precise analysis of the association between *GSTM1-* and *GSTT1-*null genotypes and endometriosis risk, additional studies with larger sample sizes and involving different ethnicities (especially African) are needed. Third, gene-environment interactions were not evaluated in this meta-analysis. Subgroup analyses based on environmental exposures were not performed because of insufficient data on such associations in all included studies.

In conclusion, this meta-analysis suggests that *GSTM1-* and *GSTT1*-null genotypes are associated with an increased risk of endometriosis. Null genotypes of *GSTM1* and *GSTT1* could act as biomarkers of endometriosis susceptibility. Larger and well-designed studies are needed to confirm these findings. Moreover, future studies should further evaluate potential gene-to-gene and gene-to-environment interactions to clarify the role of the *GSTM1* and *GSTT1* genes in endometriosis.

## Supporting Information

Checklist S1 **PRISMA meta-analysis checklist.**
(DOC)
